# Substance P Augments Chemokine Production by *Staphylococcus aureus* Infected Murine Osteoclasts

**DOI:** 10.1007/s10753-025-02280-x

**Published:** 2025-03-08

**Authors:** Sophie E. Sipprell, Quinton A. Krueger, Erin L. Mills, Ian Marriott, M. Brittany Johnson

**Affiliations:** 1 Department of Biological Sciences, University of North Carolina at Charlotte, Charlotte, NC 28223 USA; 2 Computational Intelligence for Predicting Health and Environmental Risks (CIPHER), University of North Carolina at Charlotte, Charlotte, NC 28223 USA

**Keywords:** Substance P, Osteoclasts, *Staphylococcus aureus*, Osteomyelitis, Chemokines, Inflammation

## Abstract

Staphylococcal osteomyelitis is a serious infection of the bone and joints characterized by progressive inflammatory tissue damage and leukocyte recruitment leading to net bone loss. Resident bone cells are capable of recognizing *Staphylococcus aureus* and initiating an inflammatory immune response that recruits leukocytes and alters bone homeostasis. Importantly, bone tissue is richly innervated with substance P containing nerve fibers and we have previously shown that this neuropeptide can augment the inflammatory responses of both osteoblasts and osteoclasts to *S. aureus* infection via neurokinin-1 receptors (NK-1R). Here, we have extended these studies by demonstrating that pharmacological inhibition of NK-1R ameliorates disease severity in a mouse model of staphylococcal osteomyelitis. This effect was associated with a significant reduction in leukocyte-attracting chemokine production following infection and reduced local levels of osteoclast and neutrophil activity. We then assessed the effect of *S. aureus* infection on bone-marrow derived osteoclast gene expression in the absence or presence of substance P. We determined that infection upregulates osteoclast expression of mRNAs encoding inflammatory mediators that include the neutrophil-attracting chemokines identified in vivo. Importantly, we found that, while substance P has no effect on chemokine mRNA expression in infected cells, this neuropeptide significantly increases the release of these chemokines by *S. aureus* challenged osteoclasts but not osteoblasts. Together, these data further support the ability of substance P to exacerbate inflammatory damage in staphylococcal osteomyelitis and indicate that this effect may be due, in part, to an augmentation of osteoclast immune responses that promote leukocyte recruitment.

**Supplementary Information:**

The online version contains supplementary material available at 10.1007/s10753-025-02280-x.

## Introduction

Osteomyelitis is a serious bacterial or fungal infection of the bone associated with progressive inflammatory bone loss [1, 2]. In the US there are 50,000 osteomyelitis cases a year, with *Staphylococcus aureus* being responsible for approximately 80% of all cases [3–6]. The increasing prevalence of antibiotic-resistant strains of *S. aureus*, along with anatomical restrictions and the difficulty of effectively removing all necrotic and infected bone tissue, compounds treatment and accounts for the high recurrence rate observed in osteomyelitis patients [2, 7–11]. Thus, there is an urgent need to further our understanding of the pathogenesis of staphylococcal osteomyelitis to identify alternative or combinatorial strategies to limit inflammatory bone loss.

During osteomyelitis the presence of inflammatory mediators, infiltrating leukocytes including neutrophils and monocytes, and dysregulation of bone homeostasis, result in necrotic tissue and net bone loss [1, 8]. Resident bone cells are now appreciated to recognize and respond to *S. aureus* [12–14] contributing to these disease hallmarks. Bone-forming osteoblasts and bone resorbing osteoclasts express pattern recognition receptors that recognize *S. aureus* and its bacterial components, thereby initiating production of inflammatory mediators including chemokines [12–18]. These chemokines can recruit and activate inflammatory leukocytes, such as neutrophils, further driving host-mediated inflammation and bone tissue loss [19, 20] due to the release of antimicrobial oxidative and non-oxidative components. Additionally, infiltrating leukocytes can serve as precursor cells for osteoclastogenesis, thereby promoting the excessive bone resorption observed in staphylococcal osteomyelitis [21, 22]. Indeed, leukocyte infiltration and increased osteoclast numbers are characteristic features of bone tissue biopsies from acute inflammatory osteomyelitis patients [23].

Bone tissue is richly innervated with nerve fibers containing substance P, a tachykinin that promotes neurogenic inflammation at many sites including the gut and the brain [24–30], and this neuropeptide has been reported to increase pro-osteoclastogenic responses associated with bone infection [12, 31, 32]. Importantly, we have recently demonstrated the robust and functional expression of neurokinin-1 receptors (NK-1R) for substance P by osteoblasts and osteoclasts and showed that substance P can augment the inflammatory cytokine responses of these cells to *S. aureus* infection [12]. Additionally, we determined that the presence of this neuropeptide alters the production of pro- and anti-osteoclastogenic factors by bacterially challenged bone cells and their proteolytic functions in a manner that would be anticipated to exacerbate inflammatory bone loss at sites of infection [12]. Furthermore, we demonstrated that the clinically approved NK-1R antagonist, aprepitant, attenuates local inflammatory and pro-osteoclastogenic mediator expression in an in vivo mouse model of post-traumatic staphylococcal osteomyelitis [12].

Here, we have extended these studies by demonstrating that pharmacological inhibition of NK-1R ameliorates disease severity in our murine osteomyelitis model. This effect was associated with the abolition of infection-induced increases in acute neutrophil-attracting chemokine production and reduced local levels of osteoclast and neutrophil activity. To begin to determine the mechanisms underlying these effects, we assessed the effect of *S. aureus* infection on bone-marrow derived osteoclast gene expression in the absence or presence of substance P. We determined that infection upregulates osteoclast expression of mRNAs encoding inflammatory mediators that include the neutrophil-attracting chemokines identified in vivo. Importantly, we found that while substance P has no effect on chemokine mRNA expression in infected cells, this neuropeptide significantly increases the release of these chemokines by *S. aureus* challenged osteoclasts, but not osteoblasts. Together, these data further support the ability of substance P to exacerbate inflammatory damage in staphylococcal osteomyelitis and indicate that this effect may be due, at least in part, to an augmentation of early osteoclast chemokine production that promotes neutrophil recruitment.

## Materials and Methods

### *Staphylococcus aureus* Propagation

The clinical *S. aureus* isolate, UAMS-1, was grown overnight on Luria broth (LB) agar plates at 37 °C in 5% CO_2_. *S. aureus* was cultured overnight in LB overnight on an orbital rocker at 37 °C in 5% CO_2_ and grown to mid-log phase prior to infection. The number of colony forming units (CFU/mL) was determined by spectrophotometry using a Nanodrop One spectrophotometer (ThermoFisher Scientific).

### Murine Model of Staphylococcal osteomyelitis

All protocols involving animals were approved by the Institutional Animal Care and Use Committee of the University of North Carolina at Charlotte. In the current study, we utilized a previously described [12, 13, 17, 18, 33] murine model of localized traumatic staphylococcal osteomyelitis that replicates the pathology of human post-traumatic osteomyelitis [12, 34]. A minimum sample size of 3 mice (male and female) per group were used for each in vivo experiment, with mice randomly selected for treatment groups. C57BL/6 J male and female mice, 8–13 weeks of age, received a 100 μL subcutaneous injection of either 10 mg/kg of the NK-1R antagonist, aprepitant (Selleck Chemical, MK-0869, cat# S1189) in 5% DMSO/95% corn oil, or the vehicle alone, at days −1, 0, and + 1 relative to the surgical infection. Prior to surgery and throughout the study duration mice received the analgesic, buprenorphine. Mice were anesthetized with inhalant isoflurane and the left femur was surgically exposed. The diaphysis of the femur was abraded on the anterior and posterior sides of the bone using a high-speed drill with a burred bit. *S. aureus*-containing agarose beads were applied to the abraded sections of the femurs to induce a localized infection. *S. aureus-*containing agarose beads were prepared by combining 1.4% low melt agarose cooled to 40–42 °C with 2 X 10^9^ CFU *S. aureus* and mineral oil. This mixture was swirled vigorously on ice to generate beads containing *S. aureus*. Prior to femur inoculation, agarose beads were washed with sterile 1X PBS and stored on ice. The muscle fascia was sutured and the surgical incision closed with staples. Animals were monitored for weight loss, alertness, and mobility twice daily until euthanasia. Any animals that lost more than 20% of their body weight or that displayed indicators of severe pain and distress were immediately euthanized. At 3 days post-infection, the infected and contralateral uninfected femur were isolated from each mouse. Femurs were weighed and processed by either snap freezing with liquid nitrogen followed by tissue protein extraction reagent (T-PER) isolation to obtain total cell protein isolates or fixed with 2% paraformaldehyde followed by decalcification for tissue cross-sectioning.

### Masson’s Trichrome Staining of Bone Tissue Cross Sections

Infected and contralateral uninfected femurs were isolated at 3 days post-infection and fixed with 2% paraformaldehyde for 24 h. The tissue was then placed in a decalcifying solution (0.5 M EDTA, 0.3 M NaOH, 15% glycerol, pH 7.1–7.4) for 5 days at 4 °C. Decalcified bone tissue was then placed through a series of wash buffer solutions containing 20% sucrose and 5–15% glycerol before being embedded in frozen optimal cutting tissue (OCT) compound. Bone tissue cross sections along the diaphysis of the bone were obtained using a refrigerated cryostat (Epredia) and placed on Superfrost Plus microscope slides (Fisherbrand, Cat# 1,255,015, Lot# 24,933). Masson’s trichrome staining was performed on 4 µm sections from uninfected and infected femurs according to the manufacturer’s guidelines (StatLab, Ref# KTMTR, Lot# 200,145). This procedure stains the cytoplasm, muscle, and erythrocytes red, collagen blue, and nuclei purple-black. The stained cross sections were imaged using a Leica DM IL LED microscope. For each sample, a minimum of 3 images were obtained per cross section and a range of 2–13 measurements of the cortical bone thickness was measured using Leica Microsystems (LAS X) software per image of each cross section. In addition, we have measured decreases in collagen staining intensity and homogeneity of these sections as indices of bone tissue integrity loss by quantifying the percent area within the bone tissue lacking Masson’s trichrome, indicating a loss of collagen within the bone tissue, using ImageJ software. A minimum of 3 cross sections per bone tissue sample from a minimum of 3 mice were used for quantification.

### Tartrate-Resistant Acid Phosphatase (TRAP) Staining of Bone Tissue Cross Sections

Bone tissue cross sections along the diaphysis of the bone were obtained as described above. TRAP staining was performed on 4 µm sections from uninfected and infected femurs of both treatment groups according to the manufacturer’s guidelines (APExBIO, Cat#K2606). The purple stain depicts TRAP and the green/blue counterstain reflects the nuclei. The stained cross-sections were imaged using a Leica DM IL LED microscope. For quantification, a minimum of 3 cross-sections were obtained per biological replicate (*n* = 3) with a range of 1–7 images per cross section. This range of images was determined based on folding or imperfections of the tissue cross sections. TRAP intensity was determined using color thresholding of the bone tissue, excluding the bone marrow, via ImageJ software.

### *Staphylococcusaureus* Immunohistochemical (IHC) Staining of Bone Tissue Cross-Sections

Bone tissue cross-sections along the diaphysis of the bone were obtained as described above. Tissue cross-sections were dehydrated with ethanol wash steps, followed by a quick rinse with deionized water and then a 10 min incubation in hydrogen peroxide. The sections were rinsed with 1X tris-buffered saline (TBS) and then blocked with 50 uL of 5% normal goat serum (NGS) diluted in 1X TBS for 30 min. Sections were incubated with 50 uL of primary antibody (1:200 dilution, Invitrogen, Ref#PA1-7246) overnight at 4 °C. Following wash steps of 1X TBS 3 times for 5 min, cross-sections were incubated with goat anti-rabbit IgG (H + L) Alexa Fluor Plus 555 secondary antibody (1:500 dilution, Invitrogen, Ref# A32732) for 30 min in the dark. The sections were washed again with 1X TBS 3 times for 5 min and then mounted with 20 uL ProLong diamond antifade mountant with DAPI (Invitrogen, Ref# P36971) on a coverslip. Representative whole tissue cross-sections were imaged using the ImageXpress Pico (Molecular Devices).

### Hematoxylin and Eosin (H&E) Staining of Bone Tissue Cross Sections

Bone tissue cross sections along the diaphysis of the bone were obtained as described above. H&E staining was performed on representative 4 µm sections from uninfected and infected femurs according to the manufacturer’s guidelines (Abcam, Cat# ab245880). The cytoplasm is represented by light pink while the collagen is represented as darker pink. The erythrocytes are represented as pink/red and the nuclei as blue. The representative stained cross-sections were imaged using a Leica DM IL LED microscope.

### Enzyme-Linked Immunosorbent Assays (ELISAs)

ELISAs were conducted on T-PER bone tissue protein isolates, cell culture supernatants, or cell protein isolates processed using mammalian protein extraction reagent (M-PER, Thermo Fisher) to quantify inflammatory mediator production in response to *S. aureus* infection at the indicated time points. The inflammatory immune mediators analyzed were TNF, IL-1β, CXCL1, CXCL2, CXCL5, CCL2, CCL4, CCL7, MMP-9, myeloperoxidase (MPO) and cathepsin K. A purified goat or rat anti-mouse capture antibody for TNF (Cat# DY410-05, 0.8 μg/mL), IL-1β (Cat# DY401-05, 4 μg/mL), CXCL1 (Cat# DY453-05, 2 μg/mL), CXCL2 (Cat# DY452-05, 2 μg/mL), CXCL5 (Cat# DY443, 4 μg/mL), MMP-9 (Cat#DY6718, 2 μg/mL), and MPO (Cat# DY3667, 0.8 μg/mL) and a biotinylated goat or rat anti-mouse detection antibody for TNF (Cat# 410–05, 0.0375 μg/mL), IL-1β (Cat# DY401-05, 0.5 μg/mL), CXCL1 (Cat# DY453-05, 0.05 μg/mL), CXCL2 (Cat# DY452-05, 0.075 μg/mL), CXCL5 (Cat# DY443, 0.05 μg/mL), MMP-9 (Cat#DY6718, 100 ng/mL), and MPO (Cat# DY3667, 0.05 μg/mL) were used to quantify the respective mouse protein concentrations according the manufacturer’s guidelines (R&D Systems). A purified rat anti-mouse CCL7 capture antibody (Abcam, Cat# 210,889–5, 2 μg/mL) and a biotinylated rat anti-mouse CCL7 detection antibody (Abcam, Cat# 210,889–5, 0.05 μg/mL) were utilized to quantify mouse CCL7 concentrations. Mouse cathepsin K concentrations were determined using a colorimetric ELISA kit according to manufacturer’s guidelines (Novus Biological, Cat# NBP3-00426). Dilutions of recombinant TNF, IL-1β, CXCL1, CXCL2, CXCL5, MMP-9, MPO (R&D Systems), CCL7 (Abcam), and cathepsin K (Novus Biological) were used to create standard curves for the respective proteins. The concentration of the proteins for in vivo and in vitro samples were determined by extrapolating the absorbances to the standard curve (*n* = 3–12). For T-PER bone tissue protein samples, ELISA results were normalized to the corresponding femur weight.

### Primary Murine Osteoblasts and Osteoclasts Differentiation

All protocols involving animals were approved by the Institutional Animal Care and Use Committee of the University of North Carolina at Charlotte. Whole calvaria were isolated from 2- to 3-day-old murine neonates according to established protocols in the field [12, 16, 35, 36]. We have previously demonstrated that our isolation procedure for murine osteoblasts yields cells that are 99% positive for osteocalcin, type I collagen, and alkaline phosphatase (ALP) consistent with [37–40]. At confluency, primary immature osteoblasts were plated in 6 well plates at a density of 2 X 10^5^ cells per well in alpha minimum essential medium (αMEM) supplemented with 10% fetal bovine serum (FBS), 0.1 M ascorbic acid, and 1 M β-glycerol phosphate and 1% penicillin/streptomycin at 37 °C in 5% CO_2_ atmosphere. Differentiation media was changed every other day for 10 days, and osteoblast differentiation was confirmed using an alkaline phosphatase staining kit (Abcam, Cat# ab242286) and positive staining assessed by bright field microscopy as we have previously described [12].

Primary murine osteoclasts were derived from the bone marrow of the humerus and femurs of adult male and female C57BL/6 J mice. Bone marrow cells were evenly seeded in 6-well plates and differentiated to mature osteoclasts with αMEM supplemented with 10% FBS, 1% penicillin/streptomycin, 100 ng/mL receptor activator of nuclear factor kappa-B ligand (RANKL) (Stem Cell Technology, Cat# 78,059), and 100 ng/mL macrophage colony-stimulating factor (M-CSF) (Stem Cell Technology, Cat# 78,059.1) for 5 days as previously described [12, 41]. To confirm differentiation, tartrate-resistant acid phosphatase (TRAP) staining was performed using a commercially available kit (Sigma Aldrich) according to the manufacturer’s instructions and the presence of TRAP-positive cells was confirmed using bright field microscopy [12, 38, 40, 42–44].

### *S.aureus* Infection of Osteoclasts and Osteoblasts

Thirty minutes prior to infection, primary murine osteoclasts and osteoblasts were either untreated or treated with 10 nM substance P (Sigma Aldrich, Cat# S6883-1MG). Cells were then infected with *S. aureus* at a bacteria-to-bone cell ratio of 75:1. Following incubation for 2 h at 37℃ in 5% CO_2_, the media was replaced with fresh media containing antibiotics. At 4 h, cell supernatants were collected or whole cell protein isolates were prepared using M-PER solution (ThermoFisher Scientific, Ref# 78,501), and total RNA was isolated using TRIzol.

### RNA Sequence Analysis

Primary murine osteoclasts were infected with *S. aureus* at a bacteria-to-osteoclast ratio of 75:1 (*n* = 3). At 4 h post-infection, RNA was isolated with TRIzol and purified using a GeneJET RNA Cleanup and Concentration Micro Kit according to the manufacturer’s guidelines (ThermoFisher Scientific). The Genomic Sequencing and Analysis Facility at the University of Texas at Austin prepared Tag-Seq libraries using an established method and they were sequenced using an NovaSeq S1 to generate 100 bp single end reads for 3’ end sequencing [45]. The adapters of the sequenced reads were adapter cut using cutadapt v. 2.6 [46] and the reads were quality checked using fastqc v0.11.9. The GRCm39 RefSeq transcriptome (GCF_000001635.27) was used as a reference to map the quality controlled reads using Bowtie2 v 2.4.1 [47]. Differential gene expression analysis was performed with the R package DESeq2 version 1.40.2 [48]. Significant changes in gene expression were characterized as a log_2_ fold change greater than two, and an adjusted p-value of less than 0.05 (Wald Statistic, Benjamini & Hochberg correction). Gene ontology terms for the transcriptome were annotated using Interproscan 5.60—92.0 [49]. Heatmaps were generated using the ComplexHeatmap v2.16.0 package [50]. ShinyGO v0.80 was used to determine pathway enrichment where the top 20 pathways with a False Discovery Rate (FDR) less than 0.05 were sorted by fold enrichment [51]. The raw and processed files are available in the BioProject publicly accessible repository under the identifier PRJNA1168989.

### Statistical Analysis

Data are expressed as the mean ± standard error of the mean (SEM). Commercially available software (GraphPad Prism, GraphPad Software, La Jolla, CA) was utilized to perform a two-way analysis of variance (ANOVA) with Šidák’s multiple comparisons test and a *p*-value of < 0.05 was considered statistically significant.

## Results

### Pharmacological Inhibition of the Substance P Receptor NK-1R Reduces Disease Severity in a Mouse Model of Staphylococcal Osteomyelitis

**Fig. 1:**
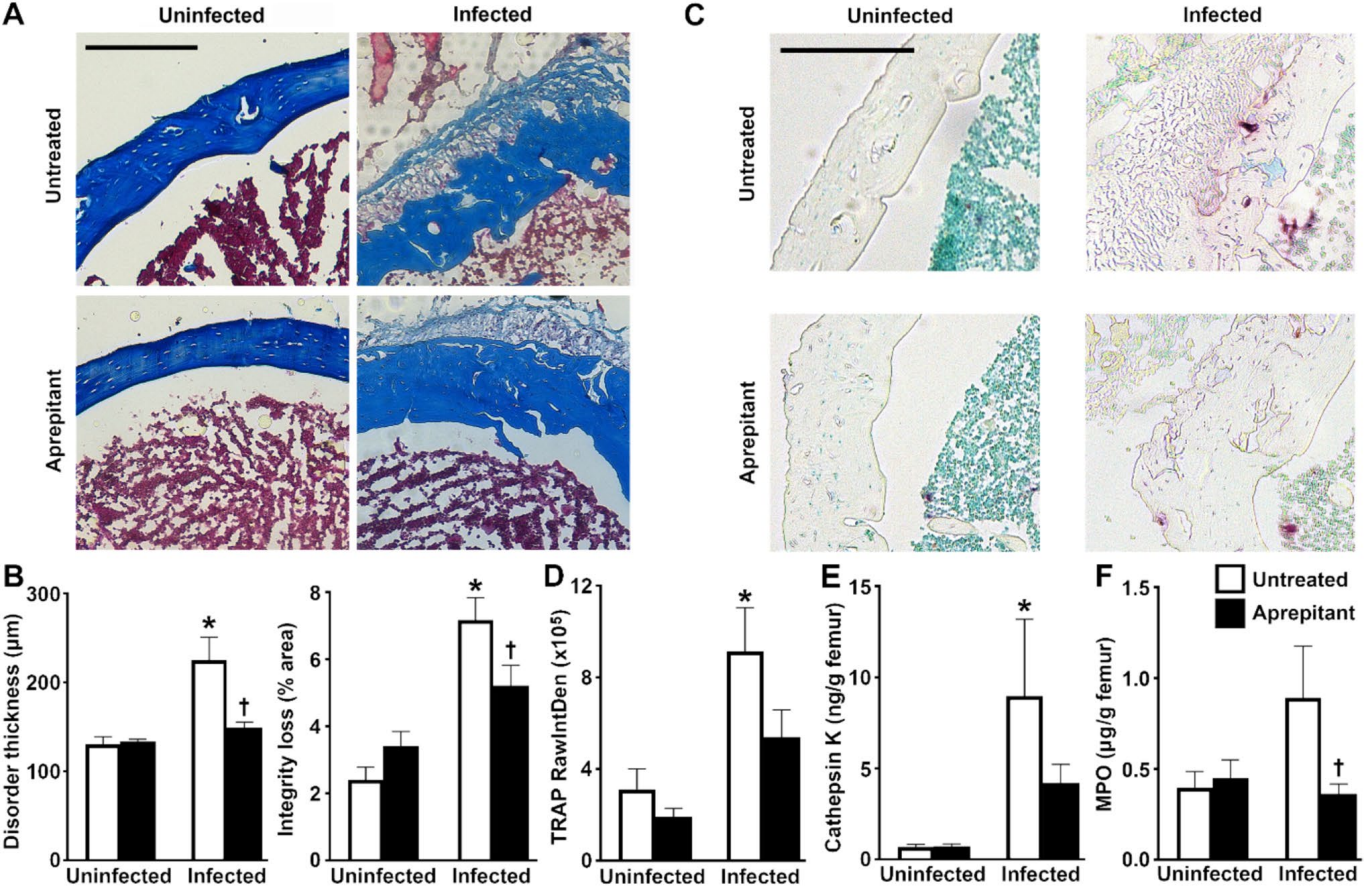
Pharmacological inhibition of the substance P receptor NK-1R reduces disease severity in a mouse model of staphylococcal osteomyelitis. Femurs were uninfected or infected with *S. aureus* and were untreated or received the NK-1R antagonist aprepitant (10 mg/kg) local to the site of bacterial administration at days −1, 0, and + 1 relative to infection. At day 3 post-infection, femurs are isolated and analyzed. Panel A: Representative sections of uninfected and infected bone tissue from untreated and aprepitant treated animals stained with Masson’s trichrome. Using this stain, the cytoplasm and erythrocytes appear red and collagen appears blue. Panel B: The cortical bone thickness at the site of abrasion and infection (Disorder thickness) and the percentage loss of Masson’s trichrome stained collagen (Integrity loss) were quantified in bone sections from each group using ImageJ software as quantitative assessments of disease severity (*n* = 3). Panel C: Representative sections of uninfected and infected bone tissue from untreated and aprepitant treated animals with TRAP staining. Using this stain, TRAP positive cells appear purple. Panel D: Osteoclast number/activity at the site of abrasion and infection were quantified according to TRAP intensity (RawIntDen) in bone sections from each group using ImageJ software. Panels E & F: Cathepsin K and MPO protein expression were assessed in protein isolates from bone tissue from each treatment group by specific capture ELISA and normalized to femur weight. Data is expressed as the mean ± SEM (*n* = 3–8). Asterisks and daggers indicate statistically significant differences compared to the corresponding uninfected contralateral femurs and the femurs of the infected untreated group, respectively (two-way ANOVA with Šidák’s multiple comparison test *p* < 0.05)

To further characterize the role of substance P/NK-1R interactions in the inflammatory damage associated with *S. aureus* infection, we have assessed the effect of pharmacological inhibition of NK-1R on disease severity in a mouse model that reproduces localized traumatic staphylococcal osteomyelitis [12, 13, 17, 18, 33]. We have previously shown that tissue damage is associated with *S. aureus* infection in this model and demonstrated that local inflammatory cytokine and chemokine production is associated with mechanical abrasion and infection, but not abrasion only [17, 18]. Here, the establishment of *S. aureus* infection was confirmed by immunohistochemistry (Figure S1). As shown in Figs. 1A and B, *S. aureus* infection caused decreased collagen staining intensity and homogeneity (integrity loss) and increased cortical bone thickness (disorder thickness) as indices of bone damage relative to uninfected contralateral femurs, consistent with hematoxylin and eosin staining (Figure S2) and prior descriptions in rodent models of osteomyelitis [22, 34, 52]. At sites of *S. aureus* infection, we also observed increased osteoclast activity as indicated by elevated levels of TRAP staining (Figs. 1C and D) and cathepsin K protein (Fig. 1E) and increased neutrophil recruitment/activation as indicated by elevated MPO (Fig. 1F), compared to contralateral control femurs, consistent with prior reports [53, 54]. Importantly, we found that local administration of the NK-1R antagonist, aprepitant, significantly attenuated infection-associated decreases in bone tissue integrity and disorder compared to vehicle treated (untreated) infected animals (Fig. 1A-B). Furthermore, aprepitant treatment significantly attenuated MPO release compared to vehicle treated infected bone tissue (Fig. 1C) and prevented the increase in cathepsin K levels in infected bone tissue (Fig. 1D). Together, these results support the contention that SP/NK-1R interactions augment inflammatory tissue damage in staphylococcal osteomyelitis, at least in part, via increased osteoclast and neutrophil activity.

### Pharmacological Inhibition of NK-1R Attenuates Bone Tissue Chemokine Production in a Mouse Model of Staphylococcal Osteomyelitis

**Fig. 2:**
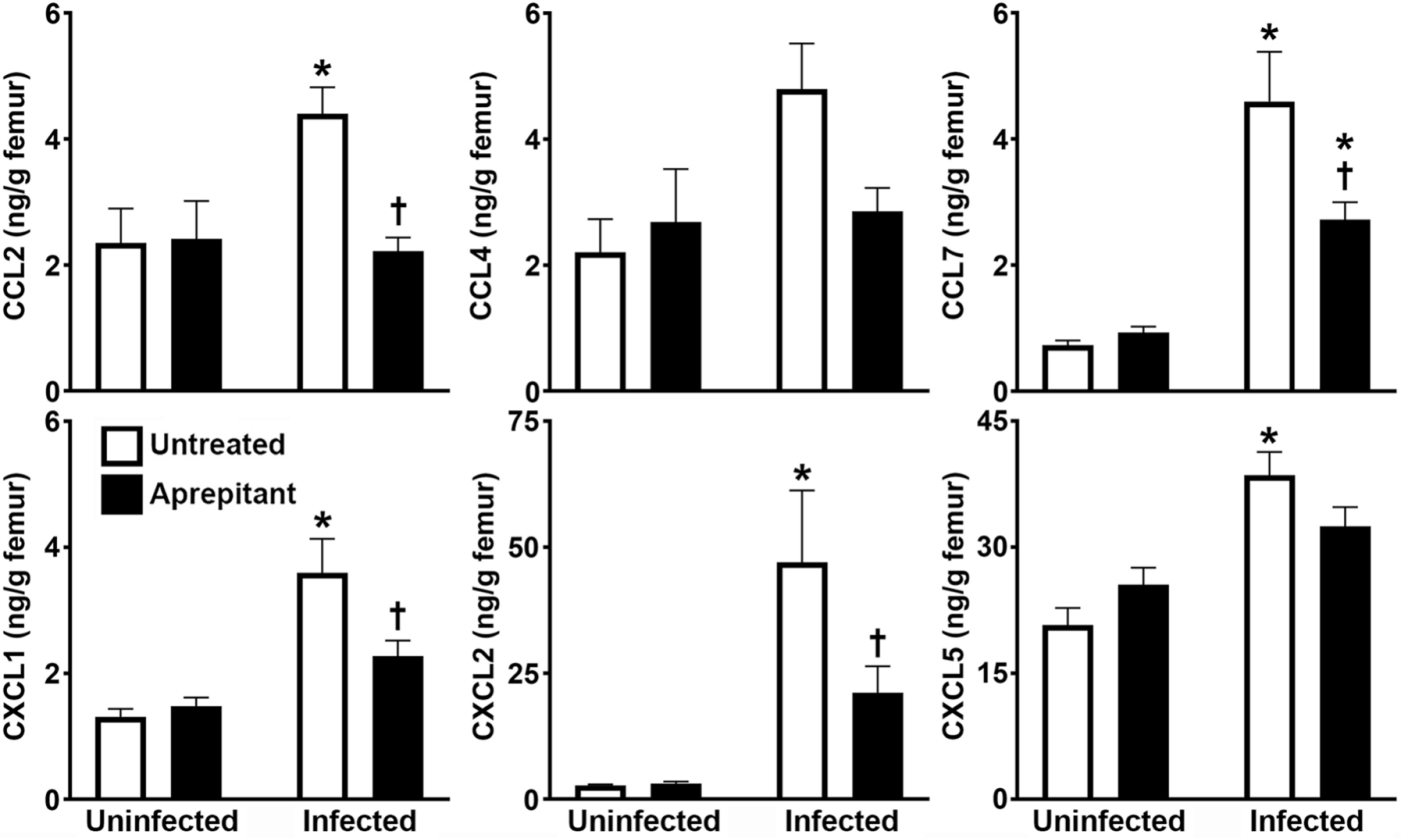
Pharmacological inhibition of NK-1R attenuates bone tissue chemokine production in a mouse model of staphylococcal osteomyelitis. Femurs were uninfected or infected with *S. aureus* and were untreated or received the NK-1R antagonist aprepitant (10 mg/kg) local to the site of bacterial administration at days −1, 0, and + 1 relative to infection. At day 3 post-infection, protein isolates were prepared from uninfected and infected bone tissue from untreated and aprepitant treated animals and were analyzed for the expression of CCL2, CCL4, CCL7, CXCL1, CXCL2, and CXCL5 by specific capture ELISA. Data is expressed as the mean ± SEM (*n* = 3–12). Asterisks and daggers indicate statistically significant differences compared to the corresponding uninfected contralateral femurs and untreated infected group femurs, respectively (two-way ANOVA with Šidák’s multiple comparison test, *p* < 0.05)

To begin to determine the mechanisms underlying the substance P-mediated increase in local MPO levels in *S. aureus* infected bone tissue, we assessed the early localized protein expression of the leukocyte-attracting chemokines, CCL2, CCL4, CCL7, CXCL1, CXCL2, and CXCL5, in the absence and presence of aprepitant treatment in our murine model of osteomyelitis. Consistent with our previously studies [12, 13], we observed a significant increase in the acute production of CCL2, CCL7, CXCL1, CXCL2, and CXCL5, in infected bone tissue, and a tendency to increase levels of CCL4 compared to the contralateral control femur (Fig. 2). Importantly, we found that aprepitant administration significantly reduced levels of CCL2, CCL7, CXCL1, and CXCL2 compared to those seen in untreated infected femurs, and a tendency to do the same for CCL4 and CXCL5 (Fig. 2). Together, these data suggest that substance P/NK-1R interactions augment local inflammatory damage in staphylococcal osteomyelitis, at least in part, by increasing the local production of chemokines capable of recruiting leukocytes to the site of infection.

### Primary Murine Osteoclasts Show Increased Expression of mRNA Encoding Proinflammatory Cytokines and Chemokines Following Challenge with *S.aureus*

**Fig. 3:**
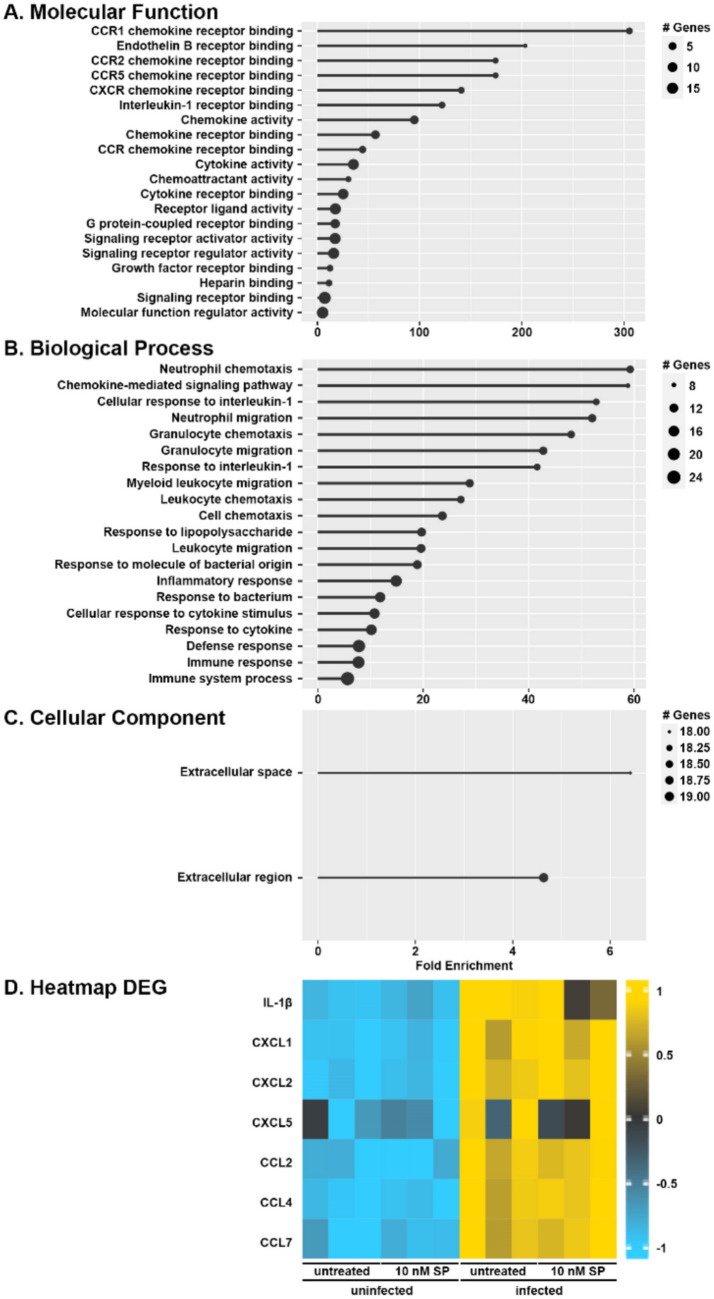
Primary murine osteoclasts increase expression of mRNA encoding proinflammatory cytokines and chemokines following challenge with *S. aureus*. Untreated or substance P treated (SP: 10 nM) bone marrow-derived osteoclasts were uninfected or infected with *S. aureus* at an MOI of 75:1 bacteria to each osteoclast and RNA was isolated at 4 h post infection. RNA Tag-Seq analysis identified 39 differentially expressed genes that displayed a twofold difference in expression (*n* = 3, adjusted *p* < 0.05). Panels A to C: Gene ontology lollipop charts displaying fold enrichment are shown for molecular function (**A**), biological processes (**B**), and cellular component location (**C**). Panel D: Heatmap displaying differential expression of genes encoding inflammatory cytokines/chemokines in untreated and substance *P* treated uninfected and *S. aureus* infected osteoclasts. The color legend indicates the z-score

To assess the role of osteoclasts in substance P-mediated inflammatory bone damage at sites of *S. aureus* infection, we explored the ability of substance P to augment the immune responses of bone marrow-derived osteoclasts. It is well established that resident bone cells contribute to immune mediator production following *S. aureus* challenge and we, and others, have shown that infected osteoblasts produce inflammatory cytokines and leukocyte recruiting chemokines [12–17, 35]. Here, we first evaluated changes in mRNA expression in murine bone marrow-derived osteoclasts following infection with *S. aureus*. As shown in Fig. 3, RNA Tag-Seq analysis identified 39 differentially expressed genes (DEGs) with a greater than log twofold change and an adjusted p-value of less than 0.05 in osteoclasts following *S. aureus* challenge (Figure S3). Our gene ontology analysis (Fig. 3A-C; Figure S4) demonstrated that infected osteoclasts display enrichment of genes associated with chemokine receptor binding and activity, as well as neutrophil chemotaxis. Of the 39 DEGs identified, there was a marked increase in the expression of mRNA encoding IL-1β, CXCL1, CXCL2, CCL2, CCL4, and CCL7, and a tendency to increase CXCL5 mRNA levels, in *S. aureus* challenged osteoclasts as early as 4 h post-infection (Fig. 3D). Interestingly, treatment with substance P failed to appreciably augment *S. aureus*-induced increases in gene expression by osteoclasts (Fig. 3D).

### Substance P augments Protein Release of Select Proinflammatory Mediators by *S.aureus*-Challenged Osteoclasts

**Fig. 4:**
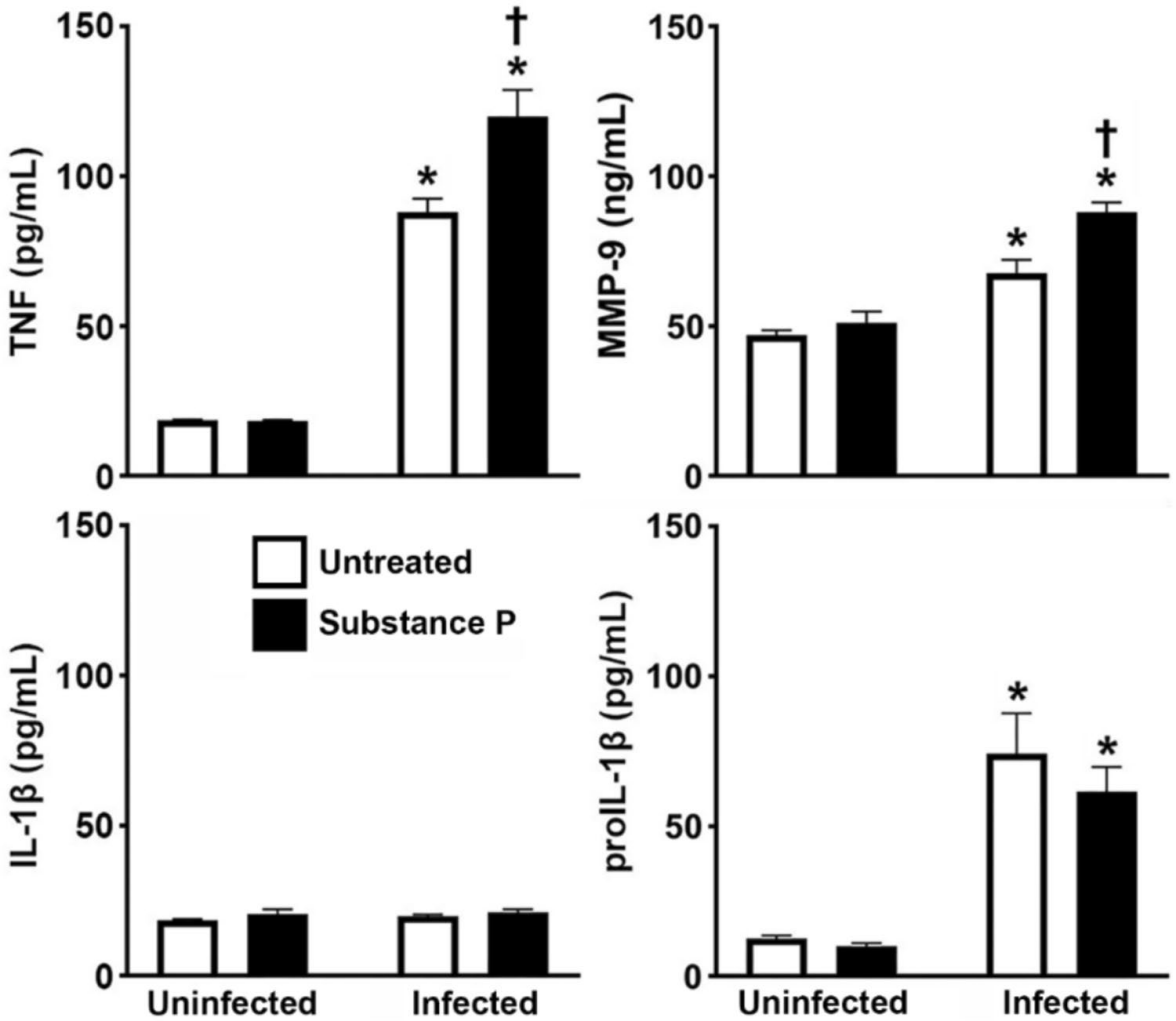
Substance P augments the production of some, but not all, proinflammatory mediators by *S. aureus* challenged osteoclasts. Untreated or substance P treated (10 nM) osteoclasts were uninfected or infected with *S. aureus* at an MOI of 75:1 bacteria to each osteoclast and the release of TNF, MMP-9, and IL-1β, and the cellular proIL-1β content was assessed at 4 h post infection by specific capture ELISA. Data is expressed as the mean ± SEM (*n* = 2–4). Asterisks indicate a statistically significant difference compared to the corresponding uninfected cells, and daggers indicate a statistically significant difference compared to untreated infected cells (two-way ANOVA with Šidák’s multiple comparison test, *p* < 0.05)

Consistent with our prior studies [12], we have found that murine osteoclasts produce TNF in response to *S. aureus* infection and showed that such production was augmented in the presence of substance P (Fig. 4) despite a lack of significant changes in mRNA expression. Similarly, no increase in MMP-9 mRNA was detected in osteoclasts following infection and yet statistically significant increases in MMP-9 protein production were detected following *S. aureus* challenge and such release was augmented following substance P treatment (Fig. 4). In contrast, we found that *S. aureus* induced increased intracellular levels of the immature form of IL-1β (proIL-1β) in osteoclasts (Fig. 4) in accordance with the observed increase in IL-1β mRNA expression at 4 h following infection (Fig. 3D). However, infection failed to elicit the release of mature IL-1β, and this is consistent with our prior reports in murine osteoblasts [55]. Furthermore, substance P failed to affect the level of expression of either intracellular proIL-1β or the mature secreted IL-1β (Fig. 4).

**Fig. 5:**
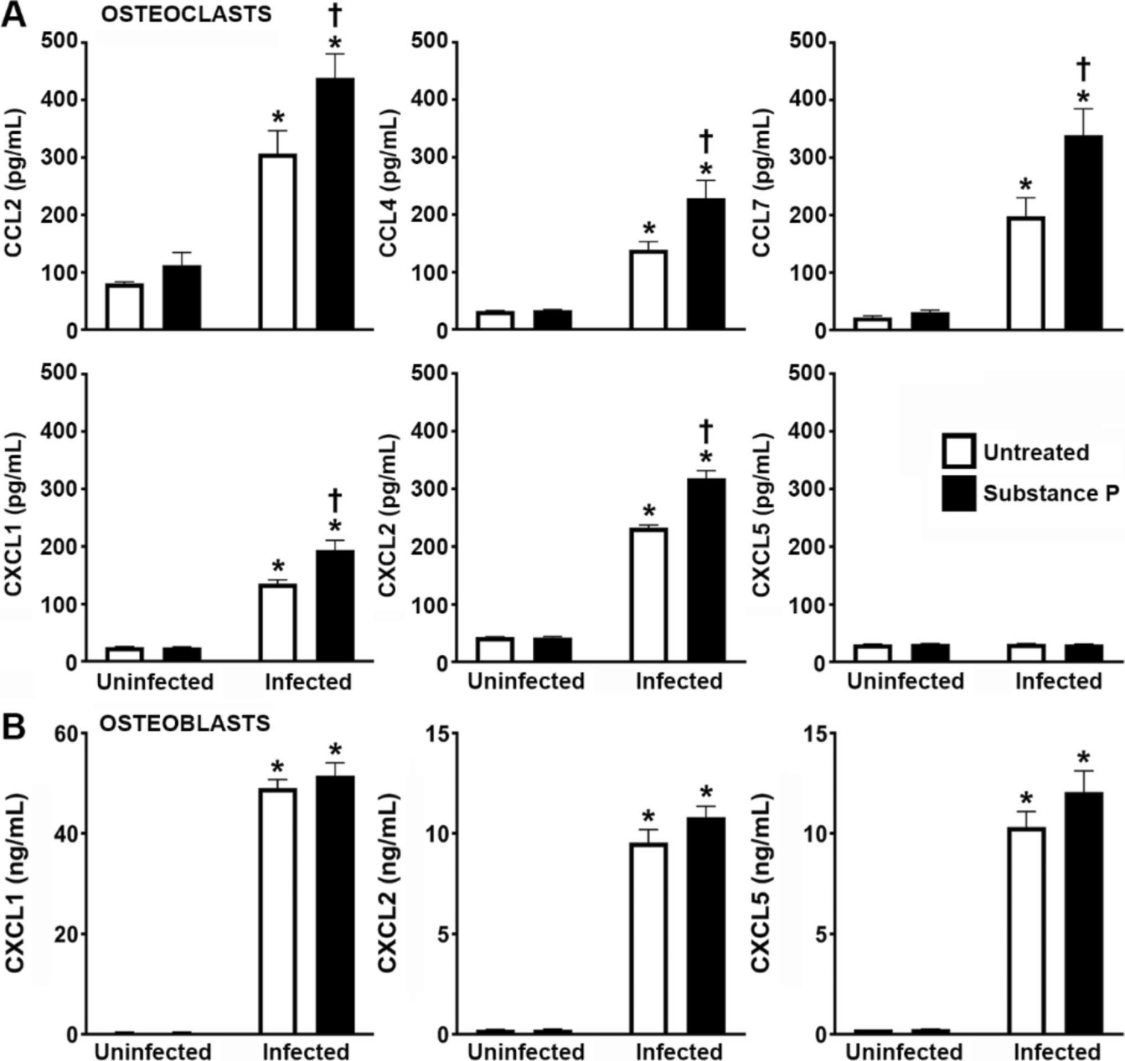
Substance P augments the production of chemokines by *S. aureus* challenged osteoclasts, but not similarly challenged osteoblasts. Untreated or substance P treated (10 nM) osteoclasts (Panel A) or osteoblasts (Panel B) were uninfected or infected with *S. aureus* at an MOI of 75:1 bacteria to each cell, and the production of CCL2, CCL4, CCL7, CXCL1, CXCL2, and CXCL5 was assessed at 4 h post infection by specific capture ELISA. Data is expressed as the mean ± SEM (*n* = 3). Asterisks indicate a statistically significant difference compared to the corresponding uninfected cells, and daggers indicate a statistically significant difference compared to untreated infected cells (Two-way ANOVA with Šidák’s multiple comparison test, *p* < 0.05)

Next, we determined whether the observed increases in the expression of mRNA encoding neutrophil-attracting chemokines by osteoclasts following *S. aureus* infection correspond with increases in protein production. As shown in Fig. 5, *S. aureus* challenge induced the release of CCL2, CCL4, CCL7, CXCL1, and CXCL2, by osteoclasts consistent with its effects on mRNA expression (Fig. 3D), but failed to elicit CXCL5 production. Importantly, the presence of substance P significantly augmented *S. aureus*-induced osteoclast CCL2, CCL4, CCL7, CXCL1, and CXCL2 production (Fig. 5A). These results contrast with our findings in osteoblasts, where infection elicited robust CXCL1, CXCL2, and CXCL5 responses that were not appreciably altered by the presence of substance P (Fig. 5B). Together, these results further demonstrate the ability of substance P to enhance the inflammatory responses of osteoclasts to *S. aureus* challenge and support the notion that this neuropeptide exacerbates inflammatory damage associated with staphylococcal osteomyelitis, at least in part, via an augmentation of the leukocyte-recruiting immune functions of these cells.

## Discussion

Inflammatory tissue damage and leukocyte recruitment are hallmarks of staphylococcal osteomyelitis [1, 8]. In agreement with previous studies [22, 52, 56], we demonstrate that *S. aureus* infection induces acute inflammatory bone tissue loss and increased disorder in a murine model of localized staphylococcal osteomyelitis. During osteomyelitis, dysregulation of bone homeostasis contributes to net bone loss and we have found increased levels of pro-osteoclastogenic factors, such as RANKL, in *S. aureus* infected bone tissue [12]. Similarly, we show in the present study that cathepsin K expression, an indicator of osteoclast activity, is also increased following *S. aureus* challenge, which may be due to the increased osteoclast numbers previously observed in infected bone tissue [52, 54].

While leukocytes such as neutrophils are important for bacterial clearance, their infiltration can directly contribute to inflammatory bone tissue damage during osteomyelitis and may even serve as an intracellular reservoir for persistent *S. aureus* infections [57]. MPO, a marker of neutrophil activation, has previously been shown to be released by neutrophils in response to *S. aureus* [58]*,* and neutrophil activity has been shown to be increased in adolescent patients with acute hematogenous osteomyelitis [59] and implant-associated models of staphylococcal osteomyelitis [52, 53]. Consistent with this, we have shown increased local levels of MPO in infected bone tissue, indicative of increases in neutrophil activity at these sites. Further evidence of an important role for infiltrating neutrophils in inflammatory bone damage includes the early increased expression of neutrophil-attracting chemokines, such as CXCL2, at osteolytic sites in patients with implant-associated osteomyelitis [56], and our prior demonstration that levels of CCL2, CCL3, CCL7, CXCL1, CXCL2, CXCL3, and CXCL5, are elevated in our murine osteomyelitis model [13, 56], a finding that has been confirmed in the present study.

Pharmacological NK-1R inhibitors, such as aprepitant, are well tolerated in human subjects and have been clinically approved for the prevention of postoperative and chemotherapy-induced emesis [60]. Importantly, we have found that the targeting of substance P/NK-1R interactions with aprepitant markedly reduced the bone integrity loss and tissue disorder associated with in vivo* S. aureus* infection. This finding is consistent with previous demonstrations of aprepitant’s ability to reduce inflammatory damage at other sites of microbial infection, such as the lung and brain [27, 61, 62]. Our data further suggests that aprepitant-mediated decreases in bone tissue damage result, at least in part, due to reductions in local osteoclast and neutrophil activity as evidenced by lower levels of cathepsin K and MPO production, respectively, in infected bone tissue. Additionally, we observed that inhibition of substance P/NK-1R interactions significantly reduced the local production of the leukocyte-recruiting chemokines, CXCL1, CXCL2, CCL2, and CCL7, in infected bone tissue. Together, these results support the exciting proposition that SP/NK-1R interactions might be targeted therapeutically to alleviate bone tissue damage during staphylococcal osteomyelitis. It should be noted that aprepitant administration did not completely abolish significant infection-induced increases in some chemokines, such as CCL7, and this may be due to incomplete NK-1R blockade, a substance P-independent component, or could even indicate substance P signaling through other neurokinin receptors such as NK-2R and NK-3R. However, such incomplete inhibition might be desirable therapeutically, as the recruitment and activation of neutrophils, and other leukocytes, is required to clear and resolve bacterial infections. Additionally, further studies are required to assess the therapeutic potential of aprepitant to alleviate bone tissue damage during chronic osteomyelitis.

To better assess the role of osteoclasts in substance P-mediated inflammatory bone damage at sites of *S. aureus* infection, we explored the ability of this neuropeptide to augment the immune responses of bone marrow-derived osteoclasts. It is now appreciated that resident bone cells are important elements in immune responses to *S. aureus* challenge and we have previously demonstrated that bone-forming osteoblasts produce an array of immune mediators following bacterial infection [14–17, 21, 63–65]. Importantly, we have previously demonstrated that both osteoblasts and osteoclasts express the substance P receptor, NK-1R, and are functionally responsive to this neuropeptide such that it augments the inflammatory/anti-osteogenic responses of these cells to *S. aureus* infection [12, 66–68]. Here, we have confirmed these prior findings with the demonstration that substance P augments the production of the proinflammatory cytokine, TNF, and the marker of osteoclast activity, MMP-9, by *S. aureus* infected osteoclasts, but failed to augment the expression of either the intracellular immature or mature secreted form of IL-1β.

With regard to an ability to attract leukocytes to sites of infection, we have previously shown that osteoblasts produce an array of chemokines capable of recruiting neutrophils including CXCL1, CXCL2, CXCL3, CXCL5, CCL2, CCL3, and CCL7 (13). Here, we show that primary bone marrow-derived osteoclasts similarly express elevated levels of mRNA encoding the chemokines, CXCL1, CXCL2, CXCL5, CCL2, CCL4, and CCL7, following *S. aureus* challenge and we subsequently confirmed the robust secretion of these leukocyte chemoattractant proteins by these cells as rapidly as four hours following infection. Importantly, we demonstrate that the presence of substance P can significantly augment the release of most of these by *S. aureus*-challenged osteoclasts but failed to do so in similarly infected osteoblasts indicating cell type specificity. However, it should be noted that the effects of substance P on osteoclast chemokine production do not appear to occur at the level mRNA expression as this neuropeptide failed to have a demonstrable effect in our RNA-Seq analyses. Furthermore, substance P did not augment the production of all the chemokines showing induced mRNA expression in osteoclasts following *S. aureus* infection as seen for CXCL5, and our inability to detect significant release of this cytokine by osteoclasts but robust production by osteoblasts suggest that the latter cell type is likely to be the predominate resident cell type responsible for the local increases in this chemokine seen in infected bone.

Taken together, the present study shows that substance P/NK-1R interactions exacerbate bone tissue integrity loss and disorder in staphylococcal osteomyelitis and indicates that this effect is associated with marked local increases in neutrophil-attracting chemokine production, and elevated osteoclast and neutrophil activity. The demonstration that osteoclasts produce many of these chemokines in response to in vitro *S. aureus* challenge, and the finding that substance P treatment significantly and specifically augments their production at the level of protein release, support an ability of this neuropeptide to exacerbate inflammatory damage in infected bone tissue that is due, at least in part, to an augmentation of neutrophil-recruiting chemokine production by osteoclasts. However, despite such suggestive findings, further in vivo experimental evidence will be required to definitively establish such a mechanism of action.

## Supplementary Information

Below is the link to the electronic supplementary material.

Supplementary file1 (DOCX 2108 KB)

## Data Availability

The raw and processed RNA sequencing files are available in the BioProject publicly accessible repository under the identifier PRJNA1168989 and the Gene Expression Omnibus (GEO) publicly accessible repository under the accession number GSE287095. Data is provided within the manuscript or supplementary files. Raw data is available upon reasonable request.
