# Supplementary material for: Substance P Augments Chemokine Production by Staphylococcus aureus Infected Murine Osteoclasts
Source: Inflammation. 2025 Mar 8;48(5):3506–18. doi: 10.1007/s10753-025-02280-x (PMC12353046; doi:10.1007/s10753-025-02280-x)
Supplement: Supplementary file 1 — Supplementary file1 (DOCX 2108 KB) [file 10753_2025_2280_MOESM1_ESM.docx]

**Supporting information for**

**Substance P augments chemokine production by *Staphylococcus aureus* infected murine osteoclasts**

Sophie E. Sipprell^1^, Quinton A. Krueger^1,2^, Erin L. Mills^1^, Ian Marriott^1^, and M. Brittany Johnson^1^

^1^Department of Biological Sciences, University of North Carolina at Charlotte, Charlotte, NC,

USA, 28223

^2^Computational Intelligence for Predicting Health and Environmental Risks (CIPHER), University of North Carolina at Charlotte, Charlotte, NC, USA 28223

*Email addresses:* ssipprel@charlotte.edu (SES), edavi138@charlotte.edu (ELM), qkrueger@charlotte.edu (QAK), imarriot@charlotte.edu (IM), and mjohn398@charlotte.edu (MBJ)

**
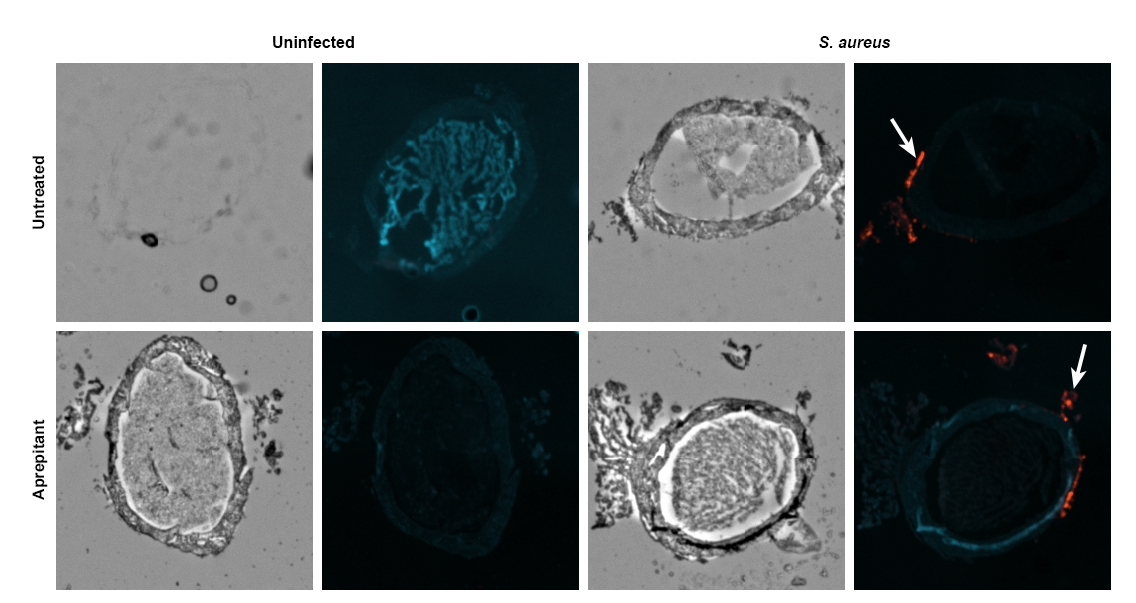
**

**SUPPLEMENTAL FIGURE 1.** Femurs were uninfected or infected with *S. aureus* and were untreated or received the NK-1R antagonist aprepitant (10 mg/kg) local to the site of bacterial administration at days -1, 0, and +1 relative to infection. At day 3 post-infection, femurs were isolated and analyzed. Representative immunohistochemical images of uninfected and *S. aureus* infected bone tissue from untreated and aprepitant treated mice. The presence of *S. aureus* is indicated in red and DAPI stained nuclei are indicated in blue. Images of bone tissue cross sections were acquired using the Molecular Devices ImageXpress Pic (4x objective).


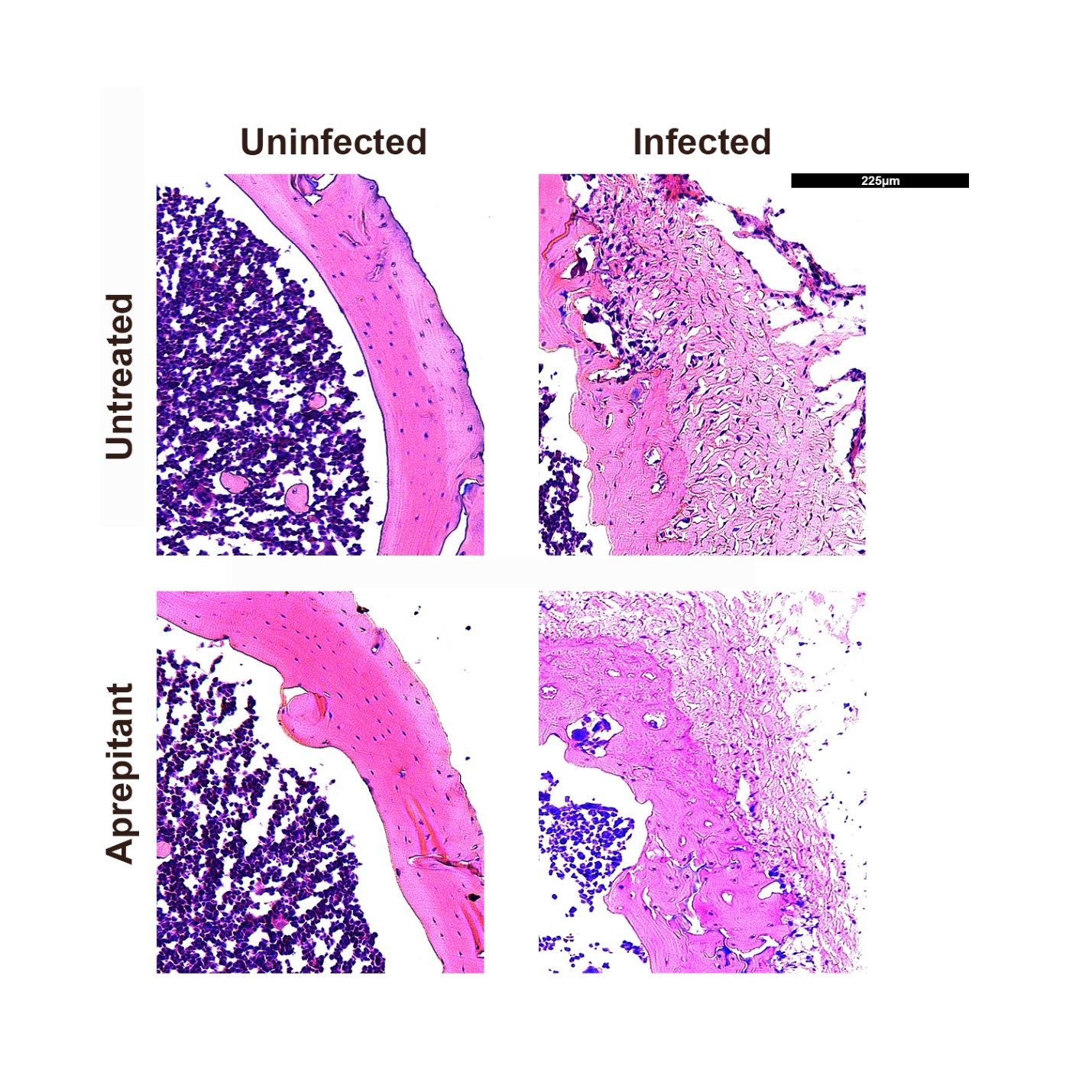


**SUPPLEMENTAL FIGURE 2.** Femurs were uninfected or infected with *S. aureus* and were untreated or received the NK-1R antagonist aprepitant (10 mg/kg) local to the site of bacterial administration at days -1, 0, and +1 relative to infection. At day 3 post-infection, femurs were isolated and analyzed. Representative bright field images of H&E staining of uninfected and infected bone tissue sections from untreated and aprepitant treated animals. Images were acquired using the Leica DM IL LED microscope (10x objective).


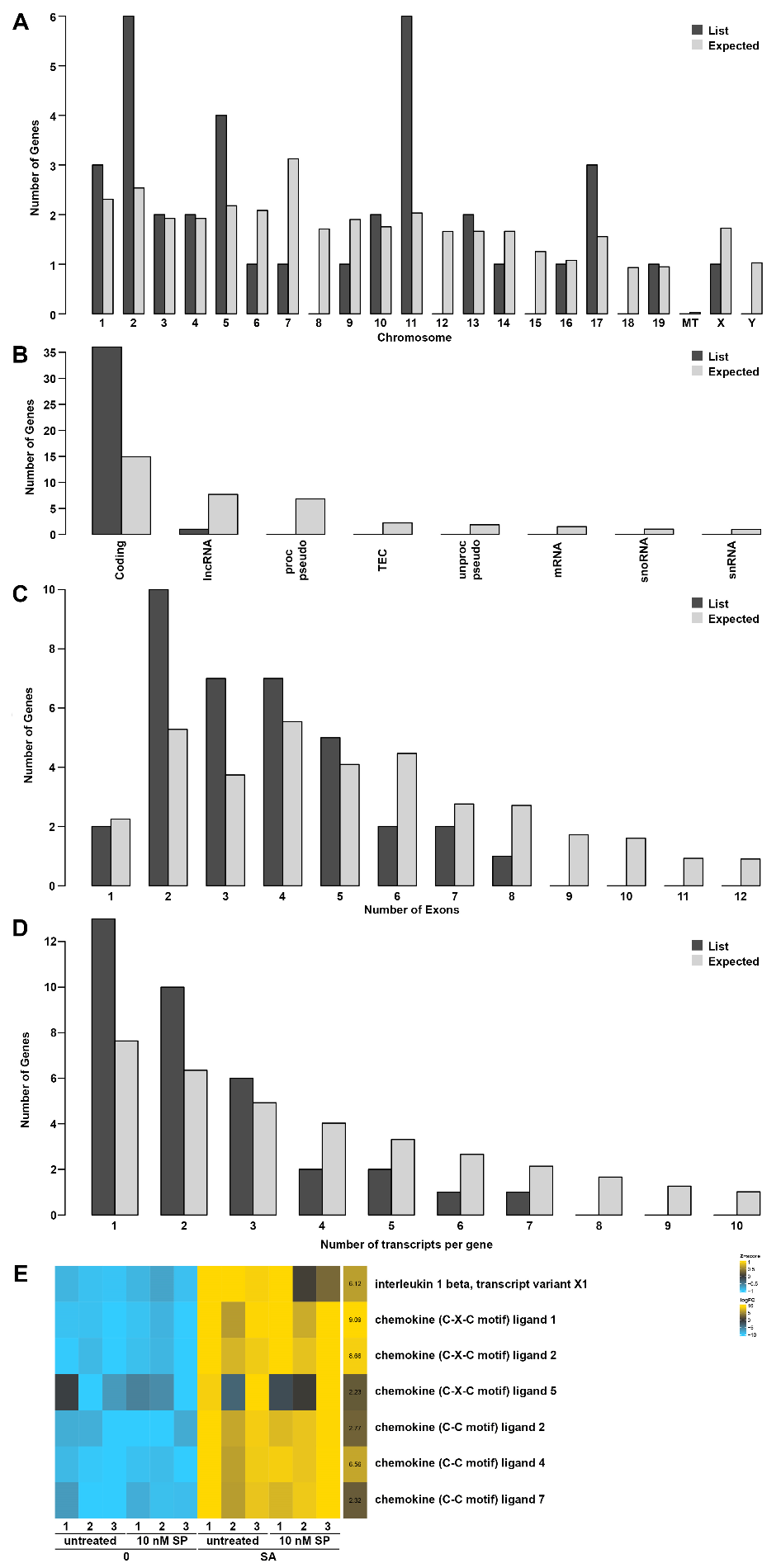


**SUPPLEMENTAL FIGURE 3.** Characteristics of differentially expression genes by *S. aureus* infected osteoclasts compared to the rest in the genome. Panel A: Distribution of query genes on the chromosomes. Panel B: Distribution by gene type. Panel C: Number of exons (coding genes only). Panel D: Number of transcript isoforms per coding gene. Panel E: A heatmap displaying differential gene expression of proinflammatory cytokines and chemokines by *S. aureus* challenged osteoclasts in the presence or absence of SP (n=3). The color legend indicates the z-score, and the average log2 fold change between uninfected and infected osteoclasts.


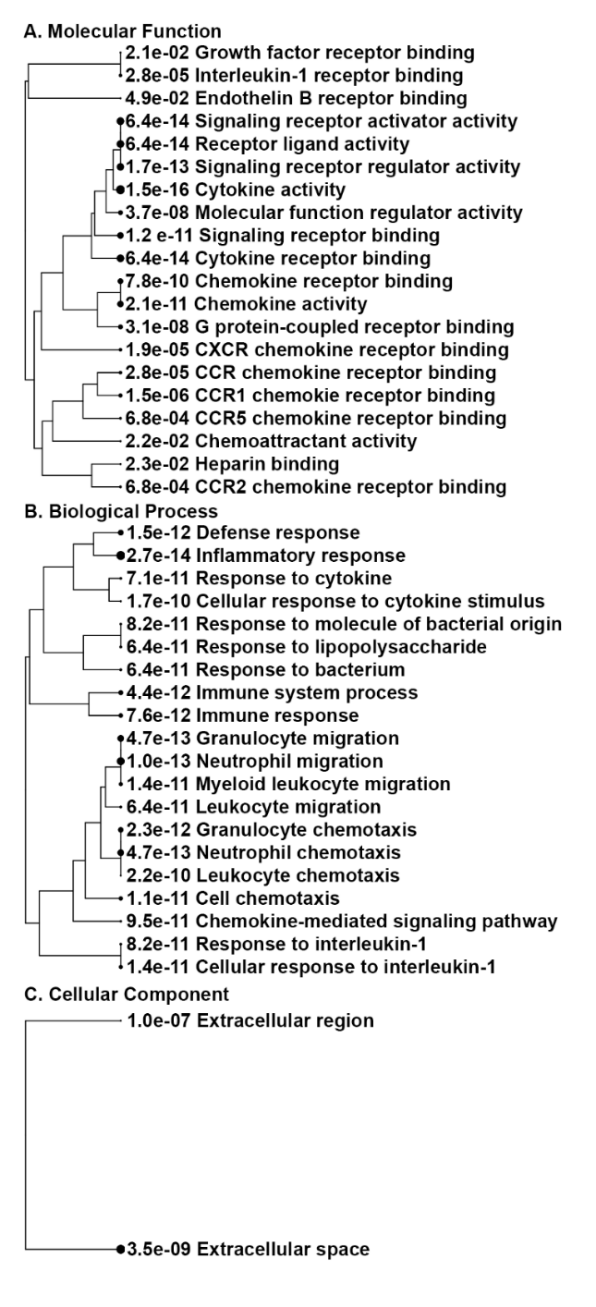


**SUPPLEMENTAL FIGURE 4.** Hierarchical clustering tree of differential gene expression summarizing the correlation among significant pathways in the molecular function (A), biological process (B), and cellular component (C), gene ontology categories expressed in osteoclasts following *S. aureus* infection. For each tree, pathways with many shared genes are clustered together and bigger dots indicate smaller p-values (number of pathways shown: 20; pathway range: 2-5000; FDR cutoff: 0.05).
